# Cortical tension regulates desmosomal morphogenesis

**DOI:** 10.3389/fcell.2022.946190

**Published:** 2022-10-04

**Authors:** Marcin Moch, Jana Schieren, Rudolf E. Leube

**Affiliations:** Institute of Molecular and Cellular Anatomy, RWTH Aachen University, Aachen, Germany

**Keywords:** Keratin, myosin II, desmosome, desmoplakin, desmoglein, desmocollin, actin, adhesion

## Abstract

Mechanical stability is a fundamental and essential property of epithelial cell sheets. It is in large part determined by cell-cell adhesion sites that are tightly integrated by the cortical cytoskeleton. An intimate crosstalk between the adherens junction-associated contractile actomyosin system and the desmosome-anchored keratin intermediate filament system is decisive for dynamic regulation of epithelial mechanics. A major question in the field is whether and in which way mechanical stress affects junctional plasticity. This is especially true for the desmosome-keratin scaffold whose role in force-sensing is virtually unknown. To examine this question, we inactivated the actomyosin system in human keratinocytes (HaCaT) and canine kidney cells (MDCK) and monitored changes in desmosomal protein turnover.

Partial inhibition of myosin II by para-nitro-blebbistatin led to a decrease of the cells' elastic modulus and to reduced desmosomal protein turnover in regions where nascent desmosomes are formed and, to a lower degree, in regions where larger, more mature desmosomes are present. Interestingly, desmosomal proteins are affected differently: a significant decrease in turnover was observed for the desmosomal plaque protein desmoplakin I (DspI), which links keratin filaments to the desmosomal core, and the transmembrane cadherin desmoglein 2 (Dsg2). On the other hand, the turnover of another type of desmosomal cadherin, desmocollin 2 (Dsc2), was not significantly altered under the tested conditions. Similarly, the turnover of the adherens junction-associated E-cadherin was not affected by the low doses of para-nitro-blebbistatin. Inhibition of actin polymerization by low dose latrunculin B treatment and of ROCK-driven actomyosin contractility by Y-27632 treatment also induced a significant decrease in desmosomal DspI turnover. Taken together, we conclude that changes in the cortical force balance affect desmosome formation and growth. Furthermore, they differentially modulate desmosomal protein turnover resulting in changes of desmosome composition. We take the observations as evidence for a hitherto unknown desmosomal mechanosensing and mechanoresponse pathway responding to an altered force balance.

## Introduction

Barrier-forming epithelia are characterized by the high degree of mechanical cohesiveness and stability. These properties rely on tight cell-cell adhesion and associated cytoskeletal networks, which form a resilient yet highly adaptable transcellular scaffold. An important architectural feature is the integration of cell-cell adhesion sites by the submembraneous cell cortex.

Two major systems can be distinguished in epithelial cells:

i) A dense layer of actomyosin is situated directly below the plasma membrane. It consists of filamentous actin and non-muscle myosin II. The myosin motor activity supports actin-dependent cortical tension (Ananthakrishnan & Ehrlicher, 2007; Chugh & Paluch, 2018). It cooperates with other components of the actin cytoskeleton, including prominent stress fibers. The actomyosin system is associated with adherens junctions (Priya & Yap, 2015), which include the classical cadherins as transmembrane receptors. They are linked to the actin system *via* linker molecules including catenins (recent review in (Rubsam et al., 2018)).
ii) A less investigated layer can be distinguished below the actomyosin layer. It consists of keratin intermediate filaments, which are anchored to desmosomal adhesions and form a web-like network structure with important mechanobiological functions (Quinlan et al., 2017; Prechova et al., 2022). A fundamental difference to the actomyosin cortex is the inability of the keratin intermediate filament network to actively generate force. But keratin filaments exhibit unique mechanical properties, notably a very high degree of flexibility and extensibility which they share with other intermediate filament types (Block et al., 2015; Yoon & Leube, 2019). Keratin intermediate filaments are attached to desmosomal cadherins of the desmoglein (Dsg) and desmocollin (Dsc) type *via* desmoplakin (Dsp). In addition, several linker molecules, including plakophilins and plakoglobin (also referred to as *γ*-catenin), connect the desmosomal components forming an electron dense plaque (recent review in (Mohammed & Chidgey, 2021; Muller et al., 2021; Hegazy et al., 2022)). The cortical keratin intermediate filaments are part of a rim and spokes system consisting of radial spokes in the cytoplasm that are connected to the submembraneous cortical rim, which interconnects desmosomes (Quinlan et al., 2017). Rim and spoke components can be generated from nascent desmosomes (Moch et al., 2019).

Evidence for a crosstalk between both the actomyosin-adherens junction and keratin-desmosome systems has accumulated over the years (Green et al., 1987; Godsel et al., 2010; Rubsam et al., 2018). Recent work has furthermore provided evidence for the functional relevance of the crosstalk to achieve mechanical homeostasis and responsiveness (Broussard et al., 2017; Prechova et al., 2022). Exactly, how changes in the mechanical environment are sensed and translated into cellular responses is less well understood. This is particularly true for the keratin-desmosome scaffold. To address this question, we decreased the intrinsic actomyosin-dependent tension by low dose treatment with the myosin II inhibitor para-nitro-blebbistatin and examined changes in desmosomal protein turnover. We observe a desmosomal protein-specific response and take this as evidence for a mechanosensing pathway that determines desmosomal protein composition and may thereby affect the mechanical tissue properties.

## Materials and methods

### Cell culture and drugs

Immortalized human HaCaT keratinocytes were kindly provided by Dr. Petra Boukamp (Boukamp et al., 1988). They were used to generate the keratin 5-YFP-expressing cell clone B10 (Moch et al., 2013). Madin-Darby canine kidney MDCK cells were obtained from the German Cancer Research Center (DKFZ; Heidelberg; *cf.* (Windoffer et al., 2022)). The cells were grown at 37 °C in a 5% CO_2_ humidified atmosphere and Dulbecco’s Modified Eagle’s Medium (DMEM) with l-alanyl-glutamine (Sigma-Aldrich, St. Louis, MO, United States of America) and 10% (v/v) fetal bovine serum (FBS; SeraPlus from PAN Biotech, Aidenbach, Germany). For passaging, cells were washed and incubated for 15 min at 37 °C in phosphate-buffered saline (PBS) without Ca^2+^/Mg^2+^ (Sigma-Aldrich). Thereafter, the cells were treated for 5–10 min with 0.05% trypsin (Genaxxon bioscience, Ulm, Germany) at 37 °C. Confluent cells were passaged once a week and were seeded at a concentration of 40,000–60,000 cells/cm^2^ in 6 ml cell culture medium in 25 cm^2^ cell culture flasks (Greiner Bio-One, Frickenhausen, Germany). For experiments, cells were grown in 35-mm diameter dishes at a concentration of ≈100,000 cells/cm^2^ (HaCaT) and 35,000 cells/cm^2^ (MDCK) with 2 ml cell culture medium. For HaCaT cells, surfaces were pre-coated with laminin 332-rich matrix from 804G rat cells as described (Langhofer et al., 1993; Moch et al., 2013). For MDCK cells, surfaces were pre-coated with 5 μg/cm^2^ human fibronectin (Sigma-Aldrich). Cells were transfected 1 day prior to experiments with 4–5 µg of plasmid DNA and 1.5 µl Xfect in a total volume of 100 µl Xfect reaction buffer (Takara Bio, Kusatsu, Shiga, Japan) per 2 ml of cell culture medium. Latrunculin B (AdipoGen, Liestal, Switzerland) and para-nitro-blebbistatin (Optopharma, Budapest, Hungary) were dissolved in pure dimethyl sulfoxide (DMSO; Sigma-Aldrich) at a concentration of 1 and 2 mM, respectively. Y-27632 (Stemcell Technologies, Vancouver, Canada) was dissolved in PBS at a concentration of 20 mM. Aliquots were stored at −20°C, and thawed aliquots were used within 1 day. When not stated otherwise, the final DMSO concentration after addition of para-nitro-blebbistatin, latrunculin B or in corresponding controls was 0.5% (v/v). Experiments were performed in 25 mM 4-(2-hydroxyethyl)-1-piperazineethanesulfonic acid-buffered DMEM without phenol red (Life Technologies, Carlsbad, CA, United States) supplemented with 2% (v/v) FBS. When not otherwise stated, cells were preincubated for 30–120 min with drug-containing or control medium prior to experiments.

### Plasmids

Plasmid DspI-GFP (p928) coding for human desmoplakin I fused to GFP was a kind gift from Kathy Green (Godsel et al., 2005). The generation of Dsc2-GFP and Dsc2-mCerulean coding for human Dsc2 (GenBank id: BC063291.1) fused to GFP or mCerulean, respectively, has been described (Windoffer et al., 2002; Moch et al., 2019). Cloning of Dsg2-GFP coding for human Dsg2 (gift from Stephan Schäfer) fused to GFP was also described previously (Moch et al., 2019). This plasmid contains the human Dsg2 cDNA (GenBank id: BC099655.3) but lacks the codons for the carboxyterminal amino acids QHSYS. E-cadherin-GFP plasmid encodes the human version of E-cadherin (GenBank id: NM_004360.5) with GFP at its C-terminus and 5′-CCGCGG-3′ nucleotides in the linker region. The N-terminus of GFP, that is encoded by 5′-ATGGTG-3′, is not present. mApple-Actin was a gift from James Nelson (Stanford University, Stanford, CA, United States).

### Measurement of elastic modulus

Elastic material properties at the cell border were measured with a Chiaro Nanoindenter equipped with an OP1550 interferometer (Optics11 Life, Amsterdam, Netherlands). The set-up was mounted on an inverted Axio Observer seven microscope (Carl Zeiss, Jena, Germany). All experiments were performed at 37°C in an incubation chamber within 30–90 min after addition of para-nitro-blebbistatin or control medium. Ferrule-top force sensors with cantilever stiffness between 0.18 and 0.29 N/m and spherical tip radius between 9.0 and 9.5 µm were used for the experiments. The cantilever was placed on the border between two flat cells at the edge of a given cell colony, as determined by bright-field microscopy. The automated “find surface” setting was chosen to approach the cell surface, followed by an indentation-controlled measurement profile of 2 s approach time to reach the final depth of 350 nm, a holding time of 1 and 2 s of retraction. In this mode, the indentation depth was controlled in real-time by refreshing the piezo stroke accordingly to feedback from the cantilever. Measurements were analyzed with DataViewer V2.4.0 software from Optics11. Since the indentation depth was considerably smaller than the indenter tip size, the Hertzian model was chosen to process the data and fit the load-indentation curve:

F=43EeffRh32

with F being the load exerted by the cantilever, E_eff_ the effective Young’s Modulus (assuming cells to be incompressible, Poisson ratio pre-configured to 0.5), R the radius of the indenter tip and h the depth of indentation. The percentage of the maximal load (Pmax %) for contact point fitting was adjusted to the true contact point. Values for effective Young’s modulus were considered with *R* ^2^ ≥ 0.9.

### Immunocytochemistry and cell fixation

For immunocytochemistry, cells were grown on 18-mm diameter high-precision glass cover slips with a thickness of 170 µm (Paul Marienfeld, Lauda-Königshofen, Germany) in six-well dishes (CytoOne^®^; Starlab International, Hamburg, Germany) for 2 days (MDCK) or 3 days (HaCaT). Fixation was performed by incubation in fresh 99.9% (v/v) methanol (Alfa Aesar, Heysham, United Kingdom) for 3 min at −20°C followed by washing in PBS (Biochrom, Schaffhausen, Switzerland) at room temperature for 5 min and optional storage over night at 4°C. Alternatively, cells were fixed in pre-warmed (37°C) 4% (v/v) paraformaldehyde (Merck, Darmstadt, Germany) in PBS (pH 7.2–7.4; adjusted with NaOH at up to 60°C) for 15 min at 37°C, then washed with PBS, and permeabilized for 3 min with 0.2% (v/v) Triton-X100 (Sigma-Aldrich) in PBS at room temperature. Primary and secondary antibodies were diluted with 1% (w/v) bovine serum albumin (BSA; SERVA, Heidelberg, Germany) in PBS. The samples were incubated with primary antibodies for 1 h, washed with PBS for 5–15 min, and incubated with secondary antibodies and 0.2 μg/ml 40,6-diamidino-2-phenylindole (DAPI; Hoffmann La Roche, Basel, Switzerland) for 40 min. Finally, cells were washed with PBS for 20 min and mono-deionized H_2_O for 30 s before mounting with Mowiol (Carl Roth, Karlsruhe, Germany) on glass slides (R. Langenbrinck, Emmendingen, Germany). The prepared samples were dried over night at 4°C and stored at the same temperature until recording within 2 weeks. Guinea pig polyclonal antibodies against DspI (DP-1) were from Progen Biotechnik (Heidelberg, Germany). Phalloidin coupled to Alexa Fluor 488 or 647 and highly cross absorbed secondary goat antibodies coupled to Alexa Fluor 488 were from Thermo Fisher Scientific (Waltham, MA, United States).

### Microscopy

Microscopical recordings were performed with a laser scanning confocal microscope (LSM 710) using Zen black 2.1 SP3 software (Carl Zeiss, Jena, Germany). The microscope was equipped with an Airyscan detector, oil immersion objective (×63/1.40-N.A, DIC M27), and a focus-shift correction system (DefiniteFocus; all from Carl Zeiss). For live-cell imaging, the microscope was pre-warmed to 37°C in an incubation chamber with 5% CO_2_. Living cells were imaged in glass-bottom dishes (12 mm glass-diameter, thickness 1.5#; MatTek, Bratislava, Slovakia) in 25 mM 4-(2-hydroxyethyl)-1-piperazineethanesulfonic acid-buffered DMEM without phenol red (Life Technologies) supplemented with 2% (v/v) FBS.

Fluorescence recovery after photobleaching (FRAP) experiments were recorded at 60 s time intervals before and after bleaching with the Airyscan detector set to standard confocal mode. The images (x = y = 33.74 µm = 512 pixel) were acquired at a pixel dwell speed of 6.27 µs, gain 850, and 16 bit-depth in three dimensions at a z-resolution of 0.65 µm. The pinhole was set to 100 µm which corresponds to two airy units for the used 460–480 and 495–550 nm emission range dual filter. For excitation of GFP the argon-ion laser (module LGK 7872 ML8) was used at 488 nm and 0.2–0.5% power. In addition, the photon multiplier was increased to the limit of safe operation. Bleaching was triggered automatically 60 s after the first z-stack within an area of 13.2 × 13.2 µm (200 × 200 pixel) for HaCaT cells or 8.45 × 16.9 µm (128 × 256 pixel) for MDCK cells. For bleaching, the laser was set to 100% power and the target area was scanned 10 times. Note that the bleaching took approximately 7–10 s resulting in a small inaccuracy between time points 0 and 1 min.

Non-FRAP experiments were recorded with all 32 PMTs of the Airyscan in the “super resolution” mode at z = 0.25 µm for immunostainings and the second-best resolution mode “resolution vs sensitivity” at z = 0.5 µm for living cells. The argon-ion laser was used at 458 nm for excitation of mCerulean, at 488 nm for GFP or Alexa488, and the photon multiplier was used at lowest setting. For excitation of mApple, a 543 nm HeNe-laser (module LGK 7786 P) was used. For excitation of DAPI, a 405 nm diode laser (laser cassette 405 cw) was used, and for excitation of Alexa 647, a 633 nm HeNe-laser (module LGK 7628-1F) was used. In immunostainings, DAPI was recorded at 420–480 nm, Alexa 488 at 460–480 nm and 495–550 nm. Live cell recordings of mApple were performed at 460–480 and 495–620 nm. In general, the detector gain was set to 850–900 for living cells and 700–850 for fixed samples. The digital gain was only increased for super resolution of living samples when necessary to a max value of 2.0. Immunostainings were scanned at second fastest speed at automatically calculated optimal resolution in unidirectional mode. Living cells for super resolution were scanned at fastest possible speed in bidirectional mode and custom resolution. Finally, the signal was processed for static samples with automatic 3D settings (including deconvolution) and for living samples with automatic 2D settings (without deconvolution). Multispectral images were acquired with a 34-channel QUASAR detector.

### Image analysis and statistical analysis

Microscopy images were processed and analyzed in the Fiji distribution of ImageJ software package (Schindelin et al., 2012; Rueden et al., 2017). The subsequent translocation of fluorescence from the unbleached cell part to the bleached region was measured as described previously (Moch et al., 2013) with two modifications. (i) The fluorescence intensity before bleaching was defined as $I^{unbleached}=I_{t=unbleached}-I_{t=0}=1=100\%$ (t = 0 corresponding to 1 min after bleaching) and the fluorescence for all subsequent time points as $I_{t=n}^{bleached}=\left(I_{t=n}-I_{t=0}\right)/I^{unbleached}$ . (ii) The measured FRAP area was smaller than the bleached area to avoid movement of unbleached desmosomes into the region of interest (see also microscopy section for parameters).

For statistical analysis, experiments were performed at least three times independently on different days except for the 10 and 20 µM para-nitro-blebbistatin treatments, which were performed on 2 days. The results for every time point and condition were combined into single datasets for each experiment. These results were tested for outliers (alpha = 0.05) with GraphPad online outlier calculator (GraphPad, San Diego, CA, United States), and, when applicable, the farthest outlier was removed. In a next step, time points were tested for Gaussian distribution with the D’Agostino and Pearson omnibus normality test and with the Shapiro-Wilk normality test using GraphPad 5.01 software (GraphPad). For FRAP experiments, we concentrated only on the last time point per condition (15 min post bleaching, t = 14) and compared it with the control. Therefore, a nonparametric (Mann-Whitney) test in GraphPad was used because not all data sets were normally distributed and the data were unpaired. Results with *p* < 0.05 were considered significant. Graphs and fitting curves (exponential rise to maximum) were made with SigmaPlot 12.0 (Inpixon, Düsseldorf, Germany). For nanoindentation experiments, all conditions were tested with the Kruskal–Wallis test before Dunn’s multiple comparison post-test was applied. Figures were prepared with Adobe Photoshop and Illustrator CS 6 (Adobe, San Jose, CA, United States of America). Movies were encoded in h.264 video format using Handbrake 1.3.3 software (https://handbrake.fr/).

## Results

### The overall organization of the actin and keratin cytoskeleton is maintained in the presence of low para-nitro-blebbistatin levels

Para-nitro-blebbistatin has been described as a non-cytotoxic and photostable alternative to blebbistatin (Kepiro et al., 2014) that can be used without noticeable side-effects at a concentration of up to 20 µM according to the manufacturer (Optopharma). However, as already reported for blebbistatin (Swift et al., 2012), we noted that para-nitro-blebbistatin has considerable autofluorescence (Supplementary Figure S1). This autofluorescence interfered with the fluorescence recordings of fluorescent desmosomal protein reporters. Thus, we observed a very strong autofluorescence of 20 µM para-nitro-blebbistatin in the detection spectrum of Dsg2-mCerulean (excitation with 456 nm light). This precluded reliable evaluation of the Dsg2-mCerulean signal (Supplementary Movie S1). On the other hand, DspI-mApple that was excited with 543 nm light in parallel did not show this problem (Supplementary Movie S1). Another serious problem that we encountered was that para-nitro-blebbistatin fluorescence increased over time reaching a peak by 15 min, which appeared to be stable (Supplementary Figure S1A). To reduce para-nitro-blebbistatin autofluorescence to acceptable levels, we lowered the drug concentration to 3 or 4 µM. In addition, we used the Argon-ion laser at 488 nm instead of 456 nm and employed GFP- instead of mCerulean-based fluorescence reporters. The emission was recorded below 550 nm, because most autofluorescence was detectable in a second peak above 547 nm (Supplementary Figure S1B).

**FIGURE 1:**
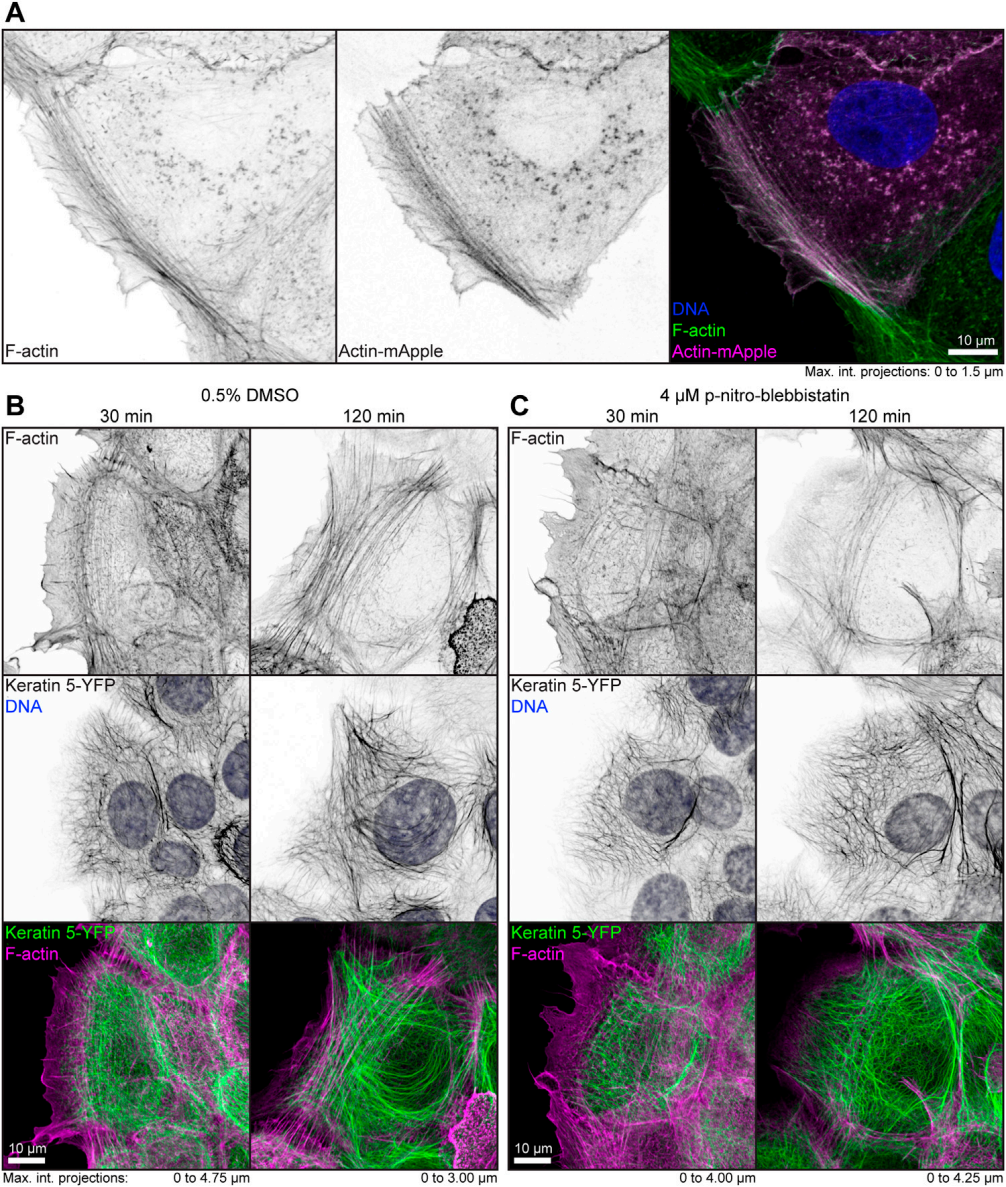
Low dose para-nitro-blebbistatin treatment does not affect the overall organization of the actin and keratin cytoskeleton. The fluorescence micrographs show maximum intensity projections of fixed HaCaT keratinocytes. **(A)** illustrates co-localization of Alexa-488-tagged phalloidin (F-Actin; left) and Actin-mApple fluorescence (middle). The overlay of both (right) reveals overlapping distribution of filamentous actin but not of cytoplasmic non-filamentous actin that is only detectable with Actin-mApple. **(B,C)** The images show the distribution of Alexa-647-tagged phalloidin (F-Actin; top) together with keratin 5-YFP and DAPI (DNA; middle; co-localization of actin and keratin in lower panel) in HaCaT B10 cells. Note that the actin and keratin cytoskeleton are not significantly affected by a 30–120 min treatment with 0.5% DMSO **(B)** and that cells remained flat and well spread even after addition of para-nitro-blebbistatin (4 µM) presenting a normal-appearing actin and keratin cytoskeleton **(C).**

To examine the effect of low-dose para-nitro-blebbistatin on the actin system, HaCaT cells were either labeled with fluorophore-coupled phalloidin or mApple-actin. Both labels detected F-actin reliably (Figure 1A). In a first set of experiments, HaCaT B10 cells were incubated with 0.5% (v/v) DMSO alone or together with 4 µM para-nitro-blebbistatin. Cells were fixed after 30 and 120 min and were subsequently stained with Alexa647 labelled phalloidin. The DMSO control presented well-developed actin stress fibers that were anchored at cell borders (Figure 1B). Cells that were treated with 4 µM para-nitro-blebbistatin retained an F-actin distribution with overall resemblance to control cells but with a slightly reduced number of actin fibers (Figure 1C). Similarly, the drug treatment had no major effect on the keratin network organization as assessed by keratin 5-YFP distribution (Figures 1B,C). We therefore concluded that the changes in the actin and keratin networks are not dramatic during 30–120 min of para-nitro-blebbistatin treatment at 4 µM and that global network rearrangement would therefore not interfere with effects of myosin inhibition.

### Myosin II inhibition decreases the elastic Young’s modulus at cell-cell contact regions

**FIGURE 2:**
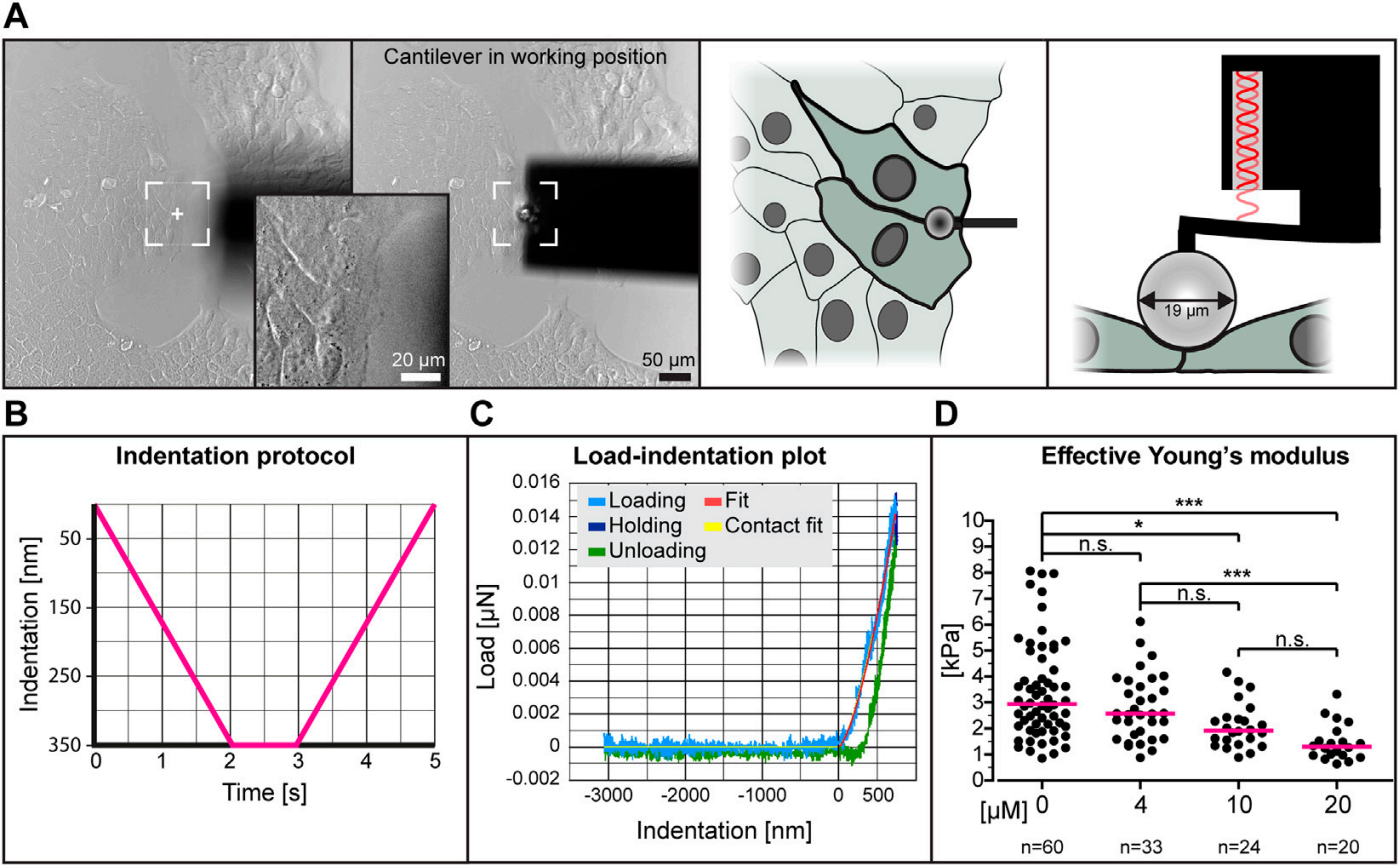
The effective Young’s modulus of HaCaT cells decreases with increased dose of para-nitro-blebbistatin. The Young’s modulus was measured with the help of a nanoindenter. **(A)** presents bright-field images at left depicting the cantilever in relation to the cells before positioning and during indentation. Note that the cantilever is placed at the border between 2 cells at the periphery of a cell colony. The schemes at right further illustrate the positioning of the cantilever from the top and the side, respectively. **(B)** The graph illustrates the indentation protocol consisting of a 2 s 350 nm indentation, 1 s holding and 2 s retraction. **(C)** The graph shows an exemplary load-indentation curve encompassing a loading (light blue), holding (dark blue) and unloading part (green). To calculate the Young’s modulus, the Hertzian model uses the fitted loading curve (red) and the contact point (yellow). **(D)** The scatter dot plot shows the effective Young’s moduli for cells treated with 0, 4, 10 and 20 µM para-nitro-blebbistatin for 30–90 min. The plot was generated by combining the medians per condition. Kruskal–Wallis test is significant with a *p* < 0.0001 and Dunn’s multiple comparison post-test shows significant differences as indicated in the graph (*p < 0.05, ***p < 0.0001).

To determine whether low dose para-nitro-blebbistatin treatment affects the elasticity of HaCaT cells, we performed nanoindentation experiments. We probed cell-cell contact regions at the edge of cell colonies (Figures 2A,B). Setting the indentation depth at 350 nm generated load-indentation plots such as that shown in Figure 2C. The effective Young’s modulus was then calculated according to the Hertzian model.

The collated data from five different experiments using different concentrations of para-nitro-blebbistatin are shown in Figure 2D. The resulting median effective Young’s modulus of untreated cells was 2.95 kPa, 1.3 kPa for 20 µM para-nitro-blebbistatin, 1.93 kPa for 10 µM para-nitro-blebbistatin and 2.57 kPa for 4 µM para-nitro-blebbistatin revealing a concentration-dependent reduction of the Young’s modulus.

### Myosin II inhibition decreases the turnover of desmoplakin I and desmoglein 2 but not of desmocollin 2 at adhering cell borders

**FIGURE 3:**
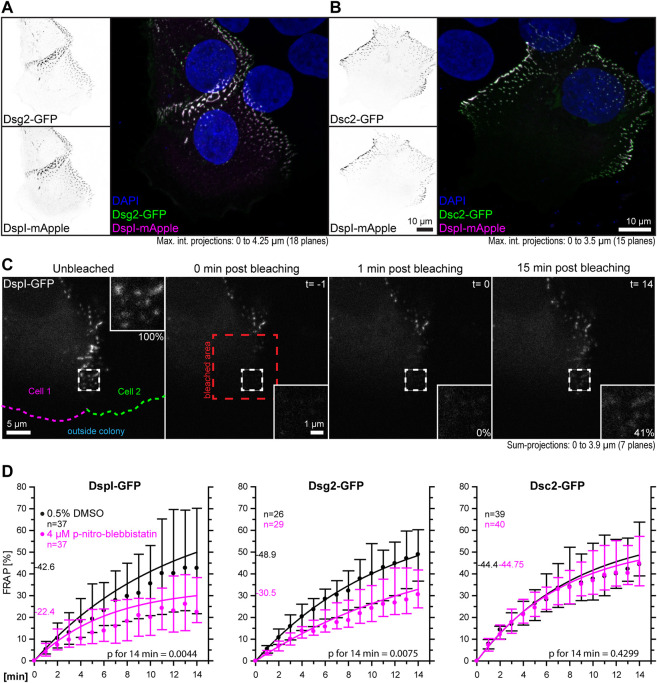
Myosin II inhibition decreases the desmosomal turnover of DspI and Dsg2 but not of Dsc2 in HaCaT keratinocytes. HaCaT cells were transfected with plasmids encoding fluorescently tagged DspI (DspI-GFP or DspI-mApple), Dsg2 (Dsg2-GFP) or Dsc2 (Dsc2-GFP). **(A,B)** presents fluorescence micrographs of cells doubly transfected to express either Dsg2-GFP or Dsc2-GFP together with DspI-mApple. Note that the fluorescent molecules co-localize in punctate structures, i. e. desmosomes that are located at borders of partially overlapping cells. **(C)** shows aspects of the workflow. It presents fluorescence images from a FRAP experiment in which cells were treated with 0.5% DMSO. The sum-projection overviews show from left to right the DspI-GFP signal in a cell just prior to bleaching, immediately, 1 and 15 min after bleaching with insets depicting the bleached region at high magnification. Fluorescence recovery measurements were started 1 minute after bleaching at which time fluorescence intensity was set as 0% and the corresponding time point was defined as t = 0. In this way, diffusion-related rapid fluorescence recovery was not taken into account for the quantitative measurements depicted in the graphs below. Aspects of this procedure are illustrated in Supplementary Movie S2 comparing solvent-treated to para-nitro-blebbistatin-treated cells. **(D)** The diagrams show time-dependent fluorescence recovery curves of DspI-GFP, Dsg2-GFP, and Dsc2-GFP that were started 1 min after bleaching (= time point 0). The experiments were performed as illustrated in **(C)**. Dots represent medians, whiskers correspond to the 25–75th percentiles, and statistical analysis was performed with a Mann-Whitney test 14 min after the measurements were started.

The goal of this study was to examine the influence of cortical tension on desmosomal polypeptide turnover. We focused on polypeptide components that are known to represent the stable moiety of desmosomes, i.e., desmosomal cadherins and desmoplakin (Fulle et al., 2021). To make sure that the dotted structures at cell-cell borders were desmosomes, we performed double-transfection experiments of different desmosomal polypeptides. As predicted, Dsg2-GFP and Dsc2-GFP localized to the same puncta as DspI-mApple (Figures 3A,B). Furthermore, we did not see any obvious differences in the distribution of the GFP- and mApple-tagged versions of DspI, both of which localized to puncta that were also labeled by anti-Dsp polyclonal antibodies, which reacted with the endogenous and exogenous Dsp versions in methanol-fixed cells (Supplementary Figure S2). Confluent, desmosome-connected HaCaT cells typically overlap. As a result, the maximum intensity projections of multiple focal planes capture not only cross-sectional views of desmosomes but also en-face views.

For desmosome turnover analyses we decided to perform FRAP analyses. To this end, cells were considered that were localized at the periphery of cell colonies. New desmosomes are formed in these regions and observations can be standardized (Moch et al., 2019). The turnover determined in this way reflects incorporation and removal of proteins into desmosomes. For simplicity’s sake, we assumed that it is mainly independent of degradation and protein synthesis. In the experiments cells were incubated with control medium or 4 µM para-nitro-blebbistatin for 30–120 min. Single transfected cells with untransfected neighbors were chosen and areas encompassing the outermost desmosomal clusters were selected for FRAP analysis. They were typically located within 10 µm of the periphery of the colonies. Larger regions of interest were bleached that contained these areas to avoid that unbleached desmosomes moved into the region that was analyzed. To capture the fluorescence of entire desmosomes, the regions of interest were scanned in three dimensions and sum-projections were prepared for analysis. Furthermore, fluorescence recovery measurements were started 1 minute after bleaching, at which time (defined as t = 0) fluorescence intensity was set as 0%. In this way, diffusion-dependent rapid fluorescence recovery, which may have occurred during the first minute after bleaching, was not taken into account. The amount of fluorescence recovery was then quantified once a minute for 14 min and compared to fluorescence recorded just prior to bleaching (Figure 3C, Supplementary Movie S2). DspI-GFP turnover was significantly reduced in para-nitro-blebbistatin-treated cells. It decreased from a median of 42.6% to a median of 22.4% after 14 min (*p* = 0.0044) (Figure 3D). A similar reduction of turnover was also observed for Dsg2-GFP. It was reduced to a median of 30.5% in para-nitro-blebbistatin-treated cells in comparison to a median of 48.9% in un-treated control cells (*p* = 0.0075) (Figure 3D). In contrast, the turnover of Dsc2-GFP was not significantly reduced after 14 min in para-nitro-blebbistatin treated cells (*p* = 0.4299) and remained nearly unaffected with 44.8 vs 44.4% (Figure 3D). Time-lapse images furthermore showed that desmosomal clusters still assembled in the presence of 4 µM para-nitro-blebbistatin (Supplementary Movie S3, Supplementary Figure S3).

**FIGURE 4:**
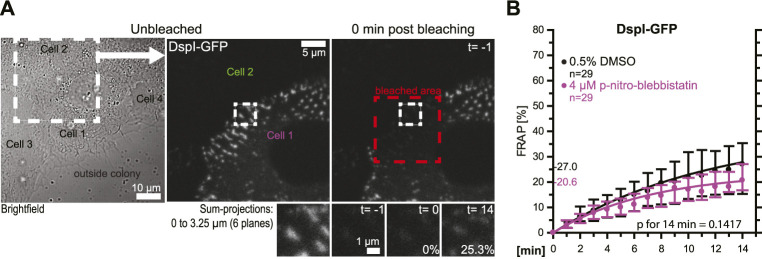
Desmosomes within a cell colony are only mildly affected by low dose myosin II inhibition. HaCaT cells were transfected with plasmids encoding fluorescently tagged DspI (DspI-GFP). **(A,B)** The micrographs and graph show results of FRAP experiments, in which DspI was bleached in desmosomes that were located toward the center of the cell colony. The microscopy pictures present from left to right an overview bright-field image and fluorescence images corresponding to the marked area before and immediately after bleaching. The region of interest prior to, immediately after, 1 and 15 min after bleaching is depicted below. The graph shows the results of multiple measurements (same denotations as in Figure 3D).

In the following experiments, turnover analyses were carried out for desmosomes that were not localized at the colony periphery (Figures 4A,B). The results revealed that these desmosomes had an overall reduced DspI-GFP turnover (median of 27.0 vs 42.6%). Treatment with 4 µM para-nitro-blebbistatin reduced the turnover further to a median of 20.6%, which was not statistically significant. Reduced baseline tension at these tightly coupled cell-cell borders may have contributed to the reduced desmosomal protein turnover and its reduced sensitivity to myosin II inhibition.

**FIGURE 5:**
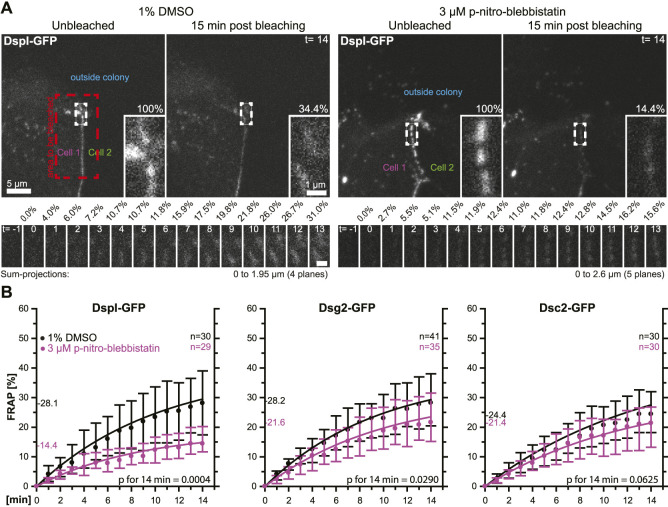
Myosin II inhibition decreases the desmosomal turnover of DspI and Dsg2 but not of Dsc2 in simple epithelial MDCK cells. MDCK cells were transfected with fluorescently tagged desmosomal proteins and fluorescence was recorded in cells at the border of colonies. **(A)** shows examples of fluorescence recordings (sum-projections) in cells incubated with control medium (left) or with 3 µM para-nitro-blebbistatin (right) just prior to bleaching and 15 min after bleaching. Note that every fluorescent cell is next to a non-transfected cell. The bleached regions of interest are presented as insets at higher magnification. Below are images of the measured regions at the intermediate time points. **(B)** The diagrams show fluorescence recovery curves of DspI-GFP, Dsg2-GFP and Dsc2-GFP that were started 1 min after bleaching. The experiments were performed as described in Figure 3C. Dots represent medians, whiskers correspond to the 25–75th percentiles, and statistical analysis was performed with a Mann-Whitney test at 14 min after the measurements were started.

In a next set of experiments, we tested whether the observed phenomena were cell type-specific. To this end, we selected simple epithelium-derived canine MDCK cells that have been used as a model to examine desmosome dynamics (Windoffer et al., 2002; Gloushankova et al., 2003). The experimental setup was identical to that used for the analysis of desmosomal protein turnover in HaCaT cells with only minor modifications. Cells were analyzed 2 days after seeding instead of 3 days after seeding, the concentration of para-nitro-blebbistatin was reduced to 3 µM and the shape of bleached regions was changed to account for the more linear arrangement of desmosomes due to the columnar cell shape of MDCK cells (Figure 5A). The results were overall confirmatory (Figure 5B). The turnover of DspI-GFP and Dsg2-GFP were significantly reduced by para-nitro-blebbistatin (*p* = 0.0004 and *p* = 0.029, respectively), whereas the turnover of Dsc2-GFP was only mildly affected (*p* = 0.0625). The base turnover of the analyzed desmosomal proteins was, however, considerably lower in MDCK cells than in HaCaT cells.

### E-cadherin turnover is not significantly affected by partial myosin II inhibition

**FIGURE 6:**
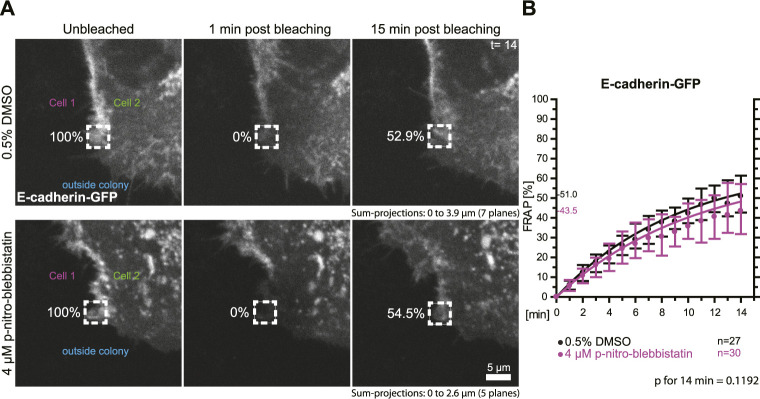
Myosin II inhibition does not significantly affect the turnover of E-cadherin at cell borders. HaCaT keratinocytes were transfected with a plasmid encoding human E-cadherin-GFP. **(A)** Regions at cell colony borders were selected for FRAP. Sum-projections of fluorescence were generated and are shown from left to right prior to, 1 and 15 min after photobleaching. The relative fluorescence measured in the regions of interest are given in %. Cells were either incubated with control medium (top) or with 4 µM para-nitro-blebbistatin (bottom). The first sum-projection shows complete E-cadherin-GFP signal in a peripheral cell that is next to a non-transfected cell directly before bleaching. **(B)** The diagram depicts the fluorescence recovery curves of E-cadherin-GFP from 1 to 15 min after photobleaching. Dots represent medians, whiskers correspond to the 25–75th percentiles, and statistical analysis was performed with a Mann-Whitney test for the last time-point.

To test whether the turnover of the adherens junction and actin-associated classical E-cadherin is affected by myosin II activity we transfected HaCaT cells with a plasmid encoding E-cadherin-GFP and performed FRAP analyses in these cells (Figure 6A). Measurements were as described for the desmosomal proteins in the preceding section. The median turnover of E-cadherin-GFP decreased slightly from a median of 51.0% in untreated control cells to a median of 43.5% in 4 µM para-nitro-blebbistatin treated HaCaT cells when measured for 14 min (Figure 6B). This decrease, however, was not significant (*p* = 0.1192). We therefore conclude that any effect on E-cadherin turnover must be weaker than that on DspI and Dsg2 and would require more sensitive or alternative detection methods.

### Depolymerization of F-actin and ROCK inhibition reduce desmoplakin I turnover in desmosomes

**FIGURE 7:**
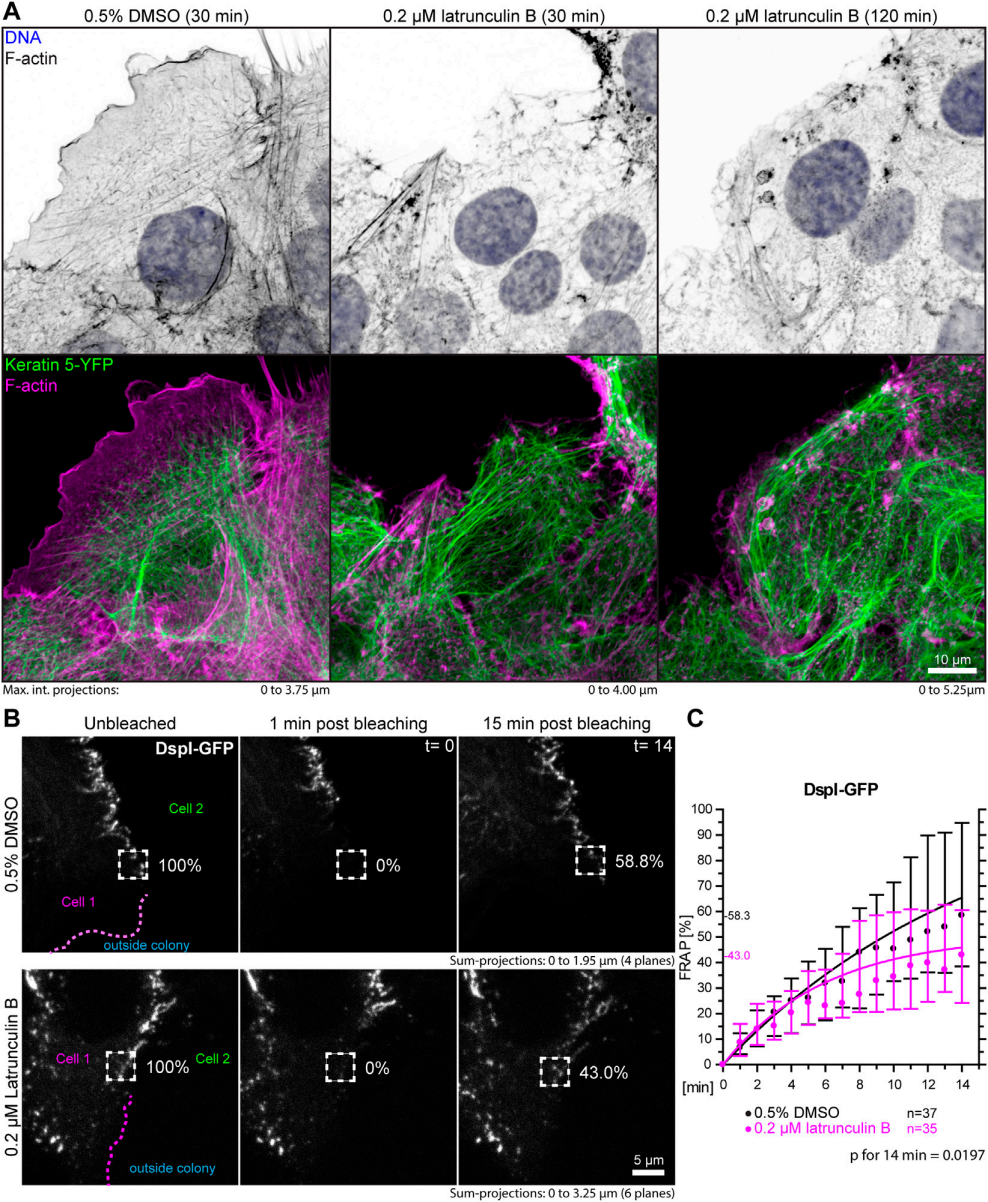
Partial depolymerization of F-actin reduces desmosomal DspI turnover. **(A)** The images show maximum intensity projections of fluorescence in fixed HaCaT keratinocytes. Actin filaments were stained with Alexa-647 tagged phalloidin (top panels) in HaCaT B10 cells that stably express keratin 5-YFP (composite images in bottom panel). The cells were incubated with control medium (0.5% DMSO; left) or 0.2 µM latrunculin B (middle and right). Note that the latrunculin B treatment leads to significant yet incomplete actin filament depletion. **(B)** shows the results of FRAP analyses. Sum-projections of confocal DspI-GFP fluorescence recordings are shown from left to right just prior to bleaching, 1 min afterwards and after 15 min in control cells (0.5% DMSO; top) and cells treated with 0.2% latrunculin B in addition (bottom). The regions of interest are demarcated by broken lines and the relative fluorescence measured in these regions are given in %. **(C)** The graph depicts the fluorescence recovery curves of DspI-GFP from 1 to 15 min after photobleaching. Dots represent medians, whiskers correspond to the 25–75th percentiles, and statistical analysis was performed with a Mann-Whitney test for the last time point.

A prediction of the blebbistatin inhibition experiments is that reduction of cortical actin would also affect desmosomal protein turnover. To test this, HaCaT cells were treated with 0.2 µM latrunculin B, which reduced cortical actin and actin stress fibers (Figure 7A, Supplementary Movie S4). At this low latrunculin concentration, the cells did not retract strongly as we previously observed for 3 µM latrunculin in HaCaT cells (Moch and Leube, 2021). Thus, we were able to minimize cell shape changes that make analyses unreliable and did not have to deal with highly mobile desmosomes, which we observed in cells with completely depolymerized F-actin. Figure 7A shows that latrunculin treatment resulted in minor changes such as a slight retraction from the cell periphery, keratin filament straightening and filament compaction, which was to be expected from previous experiments using higher latrunculin concentrations (Moch and Leube, 2021). FRAP analysis of DspI-GFP in low dose latrunculin B-treated HaCaT cells showed that desmosomal turnover is significantly reduced in comparison to control cells when measured for 14 min (43.0 *versus* 58.3%, *p* = 0.0197, Figures 7B,C).

**FIGURE 8:**
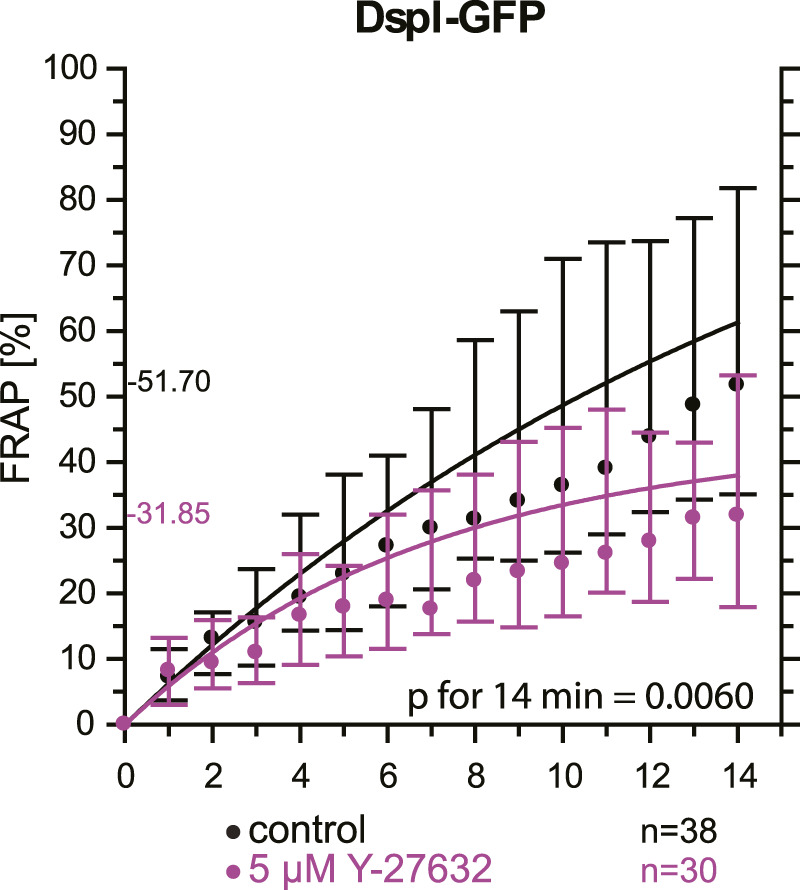
Inhibition of Rho-associated protein kinases decreases desmosomal protein turnover in HaCaT keratinocytes. The graph depicts the fluorescence recovery curves of DspI-GFP from 1 to 15 min after photobleaching in cells that were untreated (only medium change prior to experiment) or treated with 5 µM Y-27632. Dots represent medians, whiskers correspond to the 25–75th percentiles, and statistical analysis was performed with a Mann-Whitney test for the last time point.

As an alternative to actin depolymerization, we tested whether a reduction of actin-tension by inhibition of Rho-associated protein kinases ROCK1 and ROCK2 would also decrease desmosomal turnover. To this end, cells were treated with a low dose of the ROCK inhibitor Y-27632. FRAP analysis of DspI-GFP in HaCaT cells treated with 5 µM Y-27632 showed that desmosomal turnover is also significantly reduced in comparison to control cells when measured for 14 min (31.9 *versus* 51.7%, *p* = 0.006, Figure 8).

## Discussion

**FIGURE 9:**
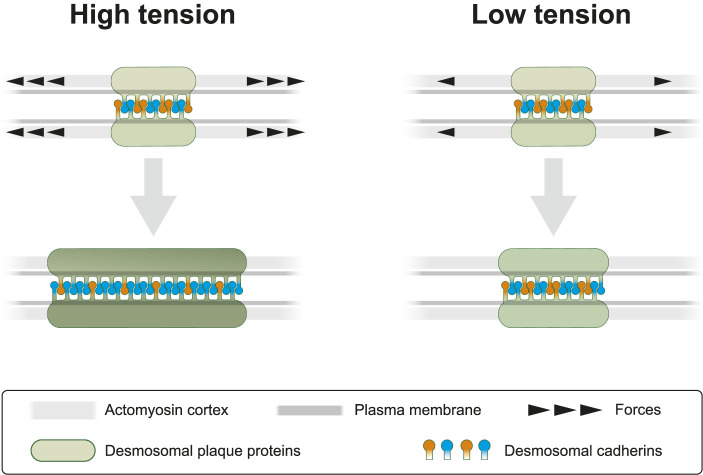
Cortical actomyosin tension regulates desmosomal morphogenesis affecting desmosome size and composition. In the present study we modulate cortical actomyosin-dependent tension by pharmacological interference. At high lateral tension desmosomal growth is supported by recruitment of desmosomal plaque proteins (green) and transmembrane proteins (orange and blue). Reduced tension negatively regulates desmosomal growth by selectively decreasing recruitment of desmosomal components. This results not only in smaller desmosomal size but also in different desmosomal composition, which is depicted as a brighter green color of the desmosomal plaque and an altered balance of desmosomal cadherins. The latter is reflected by a relative increase of “orange” cadherins (e.g., Dsc2) at low tension *versus* an increased number of “blue” cadherins (e.g., Dsg2) at high tension. We take these observations as an indication of the capacity of desmosomes to sense and respond to mechanical signals.

Desmosomes are prominent and abundant cell-cell junctions in epithelial tissues. They form, together with the associated keratin cytoskeleton, a transcellular scaffold, which is essential for epithelial tissue cohesion and mechanical stress dissipation. Yet, desmosomes are not just static structures but are subject to continuous turnover affecting all its components, albeit to different degrees (Windoffer et al., 2002; Gloushankova et al., 2003; Moch et al., 2019; Fulle et al., 2021). It is not known how this turnover is regulated and why it occurs. But it is reasonable to assume that the regulation of desmosomal protein turnover helps to adjust desmosomal size and composition to the local force balance. To test this idea, we inhibited endogenous actomyosin contractility, which is known to confer cortical tension (Ananthakrishnan & Ehrlicher, 2007; Chugh & Paluch, 2018). Our experimental results show that myosin II inhibition decreases the turnover of the desmosomal plaque protein DspI and the desmosomal cadherin Dsg2 but not the turnover of the desmosomal cadherin Dsc2 in human HaCaT keratinocytes and canine kidney epithelium derived MDCK cells. These results reveal a hitherto unknown mechanosensing response of desmosomes as summarized schematically in Figure 9. It is fully in line with the previous observation that Dsg2 experiences mechanical tension (Baddam et al., 2018) and that increased load-bearing is perceived by Dsp (Price et al., 2018).

Desmosomal protein turnover is affected by multiple pathways including (i) delivery of newly synthesized polypeptides, (ii) exchange with non-desmosomal pools and (iii) removal by endocytosis. Each of these mechanisms appears to regulate the different desmosomal components in different ways. Thus, polypeptide delivery is dependent on myosin for desmoplakin (Godsel et al., 2005; Green et al., 2010) and dependent on kinesin-1 for desmoglein 2 and kinesin-2 for desmocollin 2, respectively (Nekrasova et al., 2011). The functional importance of exchange between desmosomal and non-desmosomal pools of plasma membrane-bound desmosomal cadherins has been emphasized (Waschke & Spindler, 2014; Hiermaier et al., 2022) and likely involves lipid rafts (Resnik et al., 2011; Stahley et al., 2014). Endocytosis may affect individual desmosomal components (Delva et al., 2008; Green et al., 2010; Resnik et al., 2011; Brennan et al., 2012), desmosomal halves (Demlehner et al., 1995) or even entire desmosomes (Fulle et al., 2021). It is controlled by keratins (Kroger et al., 2013). The examples illustrate the complexity of desmosomal protein turnover and the multiple factors, which may either increase or decrease it. Detailed quantitative analyses are needed to determine the contribution of each mechanism and the involved signalling pathways to the overall context-dependent regulation of desmosomal plasticity in order to link it to desmosomal mechanosensation and mechanoresponse. Be it as it may, the observed complexity is at the root of cell type-specific and function-related desmosomal adaptation.

Tension-dependent mechanisms are attractive pathways to support desmosome formation. These pulling forces could be generated from nearby actin stress fibres that are anchored to adjacent adherens junctions or focal adhesions. In support, adherens junction formation has been shown to precede desmosome formation (Gosavi et al., 2011; Shafraz et al., 2018). These punctate contacts may exert more local tension than the closely spaced cell contacts that are encountered toward the centre of the cell colonies. There, load is shared by multiple cell-cell contacts. This force dissipation may result in smaller forces and tensional loads per desmosome. The decreased stress would then go along with decreased turnover and consequently reduced desmosome formation. Conversely, wounding would result in increased local tension through, e.g., focal adhesion-anchored actin stress fibres, creating a scenario similar to that encountered in the periphery of cell colonies, where desmosome formation is needed to mechanically couple the expanding epithelial cell layer.

A prediction of our current study is that increased changes in the force load affect desmosomal protein turnover. This hypothesis can be tested by controlled force application, e.g., in a tissue stretcher (Faust et al., 2011; Pullen et al., 2021). A precedence for load-dependent bond formation and stabilization has been provided by analyses of E-cadherin (Rakshit et al., 2012; Sivasankar, 2013). The proposed mechanobiological feedback loop of desmosomes is likely relevant for epithelial tissue morphogenesis, epithelial wound repair and tumour metastasis.

## Data Availability

The raw data supporting the conclusion of this article will be made available by the authors, without undue reservation.

## Abbreviations

BSA, bovine serum albumin; DAPI, 4′,6-diamidino-2-phenylindole; DMSO, dimethyl sulfoxideDsc2—desmocollin two Dsg2—desmoglein two; DspI, desmoplakin one; F-actin, filamentous actin; FBS, fetal bovine serum; FRAP, fluorescence recovery after photobleaching; GFP, green fluorescent protein; max. int. projection, maximum intensity projection; MDCK, Madin-Darby canine kidney (cell line); mW, milli watt; min, minute; nm, nano meter; n.s, not significant; PBS, phosphate-buffered saline; PFA, paraformaldehyde; µM, micro molar; µm, micro meter.
