# Supplementary material for: Cortical tension regulates desmosomal morphogenesis
Source: Front Cell Dev Biol. 2022 Oct 4;10:946190. doi: 10.3389/fcell.2022.946190 (PMC9577410; doi:10.3389/fcell.2022.946190)
Supplement: Supplementary file 2 [file DataSheet1.PDF]

Supplementary Material

1. Supplementary Movie Legends

Supplementary Movie 1. High-dose para-nitro-blebbistatin obscures Dsg-2-mCerulean but not DspI-mApple fluorescence. The time-lapse recording shows maximum intensity projections of the fluorescence in the 5 lower focal planes of HaCaT keratinocytes co-expressing DspI-mApple (left) and Dsg2-mCerulean (middle; merged images at right). Note the well-delineated co-distribution of DspI-mApple and Dsg2-mCerulean during the 15 min prior to the addition of 20 µM para-nitro-blebbistatin, which is difficult to track afterwards because of the strong autofluorescence of the drug, which is detectable in the cells within 2 min and subsequently accumulates to levels above signal saturation.

Supplementary Movie 2. Desmosomal protein turnover can be measured in FRAP experiments under different conditions. The two time-lapse fluorescence series show fluorescence recovery of fluorescently tagged DspI (DspI-GFP) in HaCaT keratinocytes after addition of 0.5% DMSO alone (left) and after addition of 0.5% DMSO with 4 µM para-nitro-blebbistatin (right). The movies show sum-projections of the recorded fluorescence in selected regions as described in Fig. 3C. The first frame presents the cells 60 s before bleaching was initiated and the second frame shows the situation directly after bleaching (which took 7-10 s). Fluorescence recovery measurements were started one minute after bleaching (frame 3, t=0) and fluorescence intensity was set as 0%. In this way, diffusion-related rapid fluorescence recovery was not taken into account for the quantitative measurements, which are shown in the subsequent frames for 14 minutes. Note that the cells have neighbors on the left, right and top, but not at the bottom.

Supplementary Movie 3. DspI-GFP clustering at nascent desmosomes continues in the presence of low-dose para-nitro-blebbistatin. The time-lapse recording shows the projected 7 lower planes of DspI-GFP fluorescence in a transfected HaCaT keratinocyte next to non-transfected keratinocytes. The imaged region shows cells in the periphery of a cell colony (to the right as illustrated in Fig. S3). Note that new DspI-positive puncta appear at the upper and lower left.

Supplementary Movie 4. Low dose latrunculin B and para-nitro-blebbistatin treatment affect the actin cytoskeleton differently. The time-lapse recordings of the projected lower 5 focal planes show Actin-mApple fluorescence in HaCaT keratinocytes 20 min prior to the addition of either 0.2 µM latrunculin B (left) or 4 µM para-nitro-blebbistatin (right). Note the partial restructuring of the actin cytoskeleton during the ensuing 40 min recording, which is different in both situations. Latrunculin B treatment partially disrupts the actin network whereas para-nitro-blebbistatin induces a translocation of the network toward the cell center.

2. Supplementary Figures

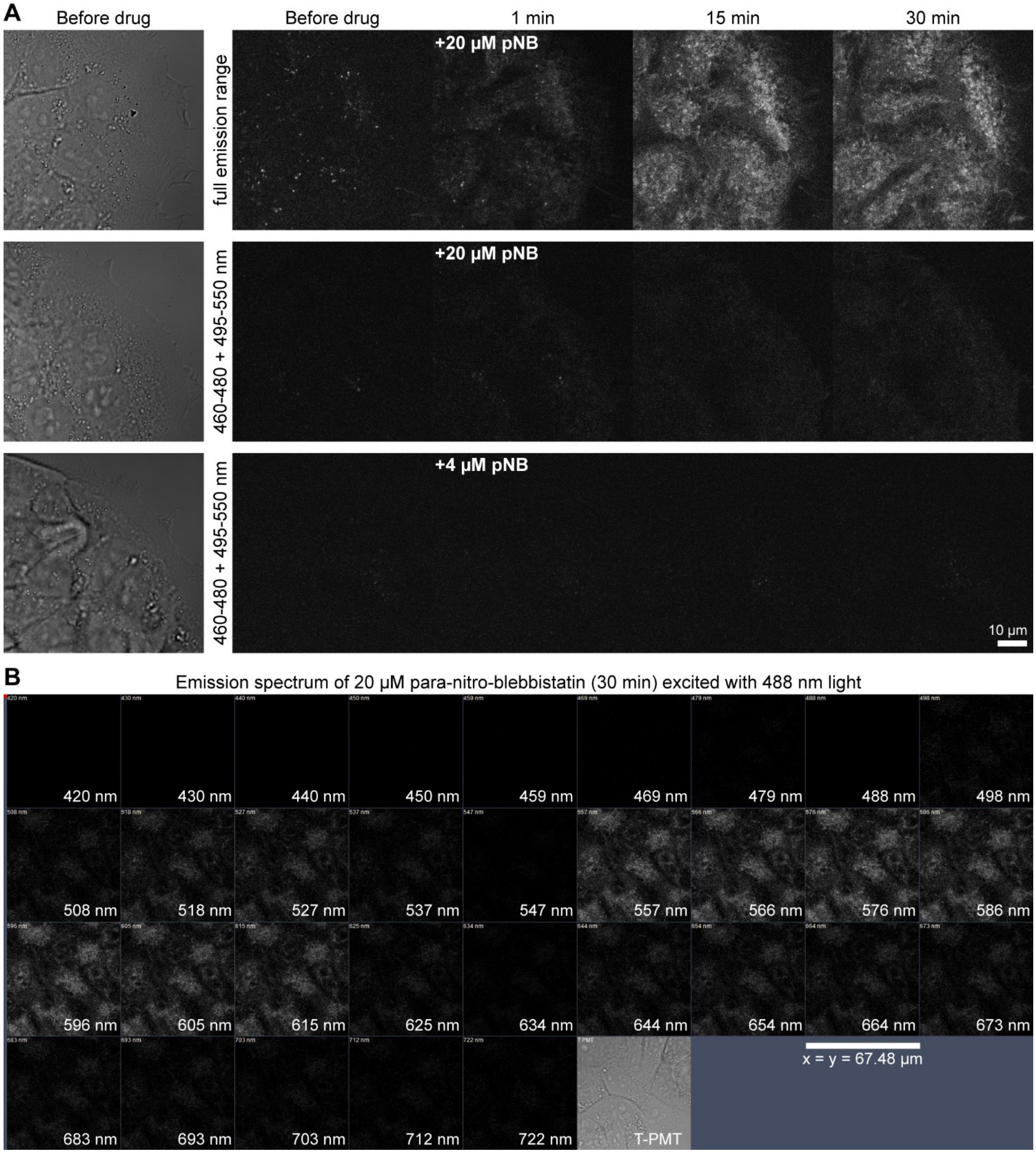

Supplementary Figure 1. Para-nitro-blebbistatin has significant autofluorescence. (A) The photomicrographs show phase contrast images (left) and corresponding fluorescence recordings of HaCaT keratinocytes before and at several time points after addition of either 20 µM or 4 µM para-

nitro-blebbistatin. Note the strong background fluorescence of 20 μM para-nitro-blebbistatin at full emission range, which increases over time (top). It is still significant at reduced emission range (i.e., 460-480 and 495-550 nm; middle), which is abolished for the most part by reduction of para-nitro-blebbistatin to 4 μM (bottom). **(B)** The fluorescence micrographs show recordings of the area depicted by bright-field microscopy (T-PMT) at different wavelengths 30 min after addition of 20 μM para-nitro-blebbistatin.

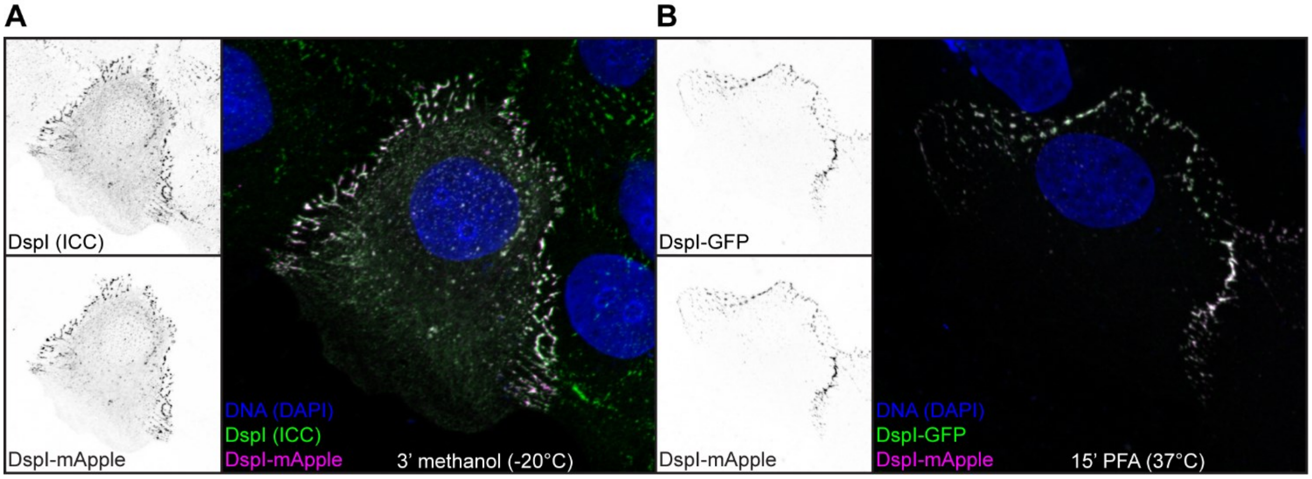

Supplementary Figure 2. Desmosomes are specifically labeled by fluorescent DspI chimeras. The fluorescence images show maximum intensity projections of HaCaT keratinocytes at the periphery of a cell colony that were either fixed with methanol for three min or with paraformaldehyde for 15 min. Note the overlap between the immunosignal (ICC) with that of the fluorescent DspI reporter DspI-mApple **(A)** and the precise co-localization of two different DspI reporters (DspI-GFP and DspI-mApple; **(B)**).

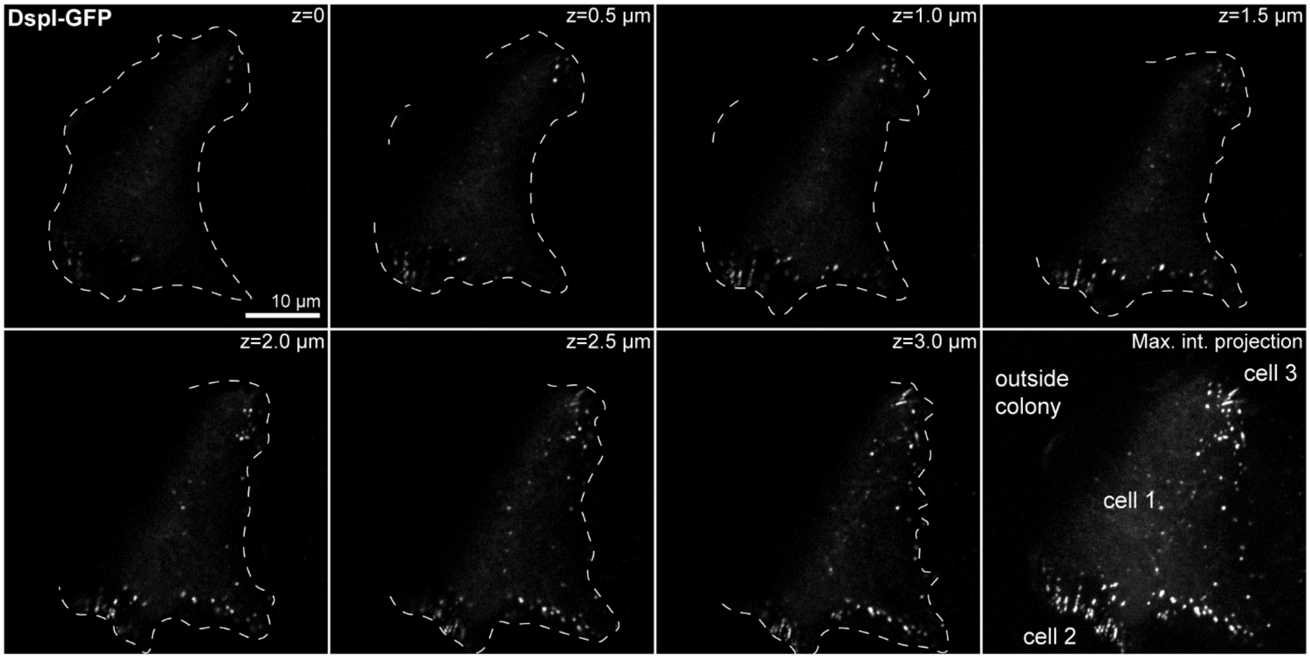

Supplementary Figure 3. Single transfected cells at the border of a cell colony provide a good overview of desmosome localization. This figure illustrates the topology of a HaCaT cell that is also

shown in Movie 2 at time point 0 min. From left to right are single confocal planes from the bottom to the middle of the cell. The cell border was demarcated (broken lines) using the cytosolic DspI-GFP fluorescence signal for orientation. The last image (lower right) shows the maximum intensity projection of all planes and the approximate localization of the neighboring cells.
